# Direct Access for Patients to Diagnostic Testing and Results Using eHealth: Systematic Review on eHealth and Diagnostics

**DOI:** 10.2196/29303

**Published:** 2022-01-12

**Authors:** Anke Versluis, Kyma Schnoor, Niels H Chavannes, Esther PWA Talboom-Kamp

**Affiliations:** 1 Department of Public Health and Primary Care Leiden University Medical Center Leiden Netherlands; 2 National eHealth Living Lab Leiden University Medical Center Leiden Netherlands; 3 Saltro Diagnostic Center Utrecht Netherlands

**Keywords:** eHealth, systematic review, diagnostic testing, home-based test, self-test

## Abstract

**Background:**

The number of people with chronic diseases and the subsequent pressure on health care is increasing. eHealth technology for diagnostic testing can contribute to more efficient health care and lower workload.

**Objective:**

This systematic review examines the available methods for direct web-based access for patients to diagnostic testing and results in the absence of a health care professional in primary care.

**Methods:**

We searched the PubMed, Embase, Web of Sciences, Cochrane Library, Emcare, and Academic Search Premier databases in August 2019 and updated in July 2021. The included studies focused on direct patient access to web-based triage leading to diagnostic testing, self-sampling or testing, or web-based communication of test results. A total of 45 studies were included. The quality was assessed using the Mixed Methods Appraisal Tool.

**Results:**

Most studies had a quantitative descriptive design and discussed a combination of services. Diagnostic test services mainly focused on sexually transmitted infections. Overall, the use was high for web-based triage (3046/5000, >50%, who used a triage booked a test), for self-sampling or self-testing kits (83%), and the result service (85%). The acceptability of the test services was high, with 81% preferring home-based testing over clinic-based testing. There was a high rate of follow-up testing or treatment after a positive test (93%).

**Conclusions:**

The results show that direct access to testing and result services had high use rates, was positively evaluated, and led to high rates of follow-up treatment. More research on cost-effectiveness is needed to determine the potential for other diseases. Direct access to diagnostic testing can lower the threshold for testing in users, potentially increase efficiency, and lower the workload in primary care.

## Introduction

### Background

As the population ages and the number of people with chronic diseases increase, the pressure on the health care system continues to rise [1,2]. This increased pressure is particularly noticeable in primary care where, over the years, the workload had already increased because of health care transformations. Primary care physicians, for example, are required to perform more preventive and complex care, work more according to evidence-based guidelines, and focus on person-centered care delivery [3,4]. Thus, physicians are required to do more in less time, and this increased workload can negatively affect the quality of patient care [4,5] and result in lower levels of job satisfaction of health care professionals (HCPs) [6,7]. Care delivery needs to be reformed to meet the needs of an aging population.

eHealth has been identified as a potential method to make health care delivery more efficient and can thereby help to decrease the workload [8,9]. eHealth can be defined as “health services and information delivered or enhanced through the Internet and related technologies*”* [10,11]. Currently, different eHealth applications are used to different extents in primary care. The advantage of eHealth applications is that health care delivery can be more efficient and can operate partially, or even completely, independent of the HCP. Gaining more insight into how eHealth is used in primary care can help to identify promising approaches that may help to lower the workload in primary care and contribute to better health care quality.

Requesting laboratory diagnostic testing, which refers to testing to determine the presence of a disease, and the communication of the results has shown promise for digitization. Indeed, eHealth technology has been applied successfully in the three stages of laboratory diagnostic testing. The first stage is *triage and advice on diagnostic testing*, where typically an HCP asks the patient a set of questions to determine whether and what diagnostic tests are relevant. An example of web-based triage was provided by Polilli et al [12], who used a web-based questionnaire (ie, triage) to determine an individual’s risk for HIV and sexually transmitted infections (STIs). On the basis of the calculated risk, individuals were automatically linked to nearby testing and counseling facilities. The second stage is the actual *testing* (eg, a blood test is performed to determine the presence of an infection). There have now been initiatives where laboratory tests can be ordered on the internet and are shipped to the individual for self-testing or self-sampling [13,14]. Self-testing refers to an approach in which individuals can collect their specimen (eg, blood) and interpret the results using a rapid diagnostic test. In self-sampling, individuals collect their specimens, but the specimen is tested elsewhere (eg, laboratory). The third stage is the *communication of test results* to the patient. A course of action is then determined based on the results. Instead of having the HCP communicate the results, it can also be communicated on the web or via an app, independent of the professional. Automated SMS text messages can be used to deliver tuberculosis testing results [15] or negative HIV test results can be automatically reported using the internet or a voicemail system. To our knowledge, a comprehensive overview of the different methods used to provide patients with direct web-based access to laboratory diagnostic testing and results is not yet available.

### Objective

The aim is to conduct a systematic review to identify and summarize the available methods for direct web-based access for participants to diagnostic testing and results in the absence of an HCP in primary care. The available reviews show promise (eg, suggesting that self-tests are acceptable and can increase the uptake and frequency of testing) [16,17], but are limited to self-sampling and self-testing and do not include other forms of digitization. Moreover, the existing reviews focus on specific populations such as men who have sex with men (MSM) [18,19] or on specific health conditions such as HIV or chlamydia [20,21]. To widen the scope, this systematic review will include studies focusing on digitization in one or more phases of laboratory diagnostic testing. Specifically, studies that focus on direct access for patients to (1) web-based triage that leads to diagnostic testing, (2) self-sampling or testing, or (3) the test results are included (or both). The review was not restricted to specific populations or health conditions. Identification and summary of possible methods for direct access to diagnostic testing and result services will help identify usable and effective methods that can potentially increase the accessibility and cost-effectiveness of health care and simultaneously reduce the workload of primary care professionals.

## Methods

The PRISMA (Preferred Reporting Items for Systematic Reviews and Meta-Analyses) guidelines for reporting systematic reviews were used [22]. The systematic review was not registered, but a strict protocol was used to search and select studies and to select data.

### Search Strategies

PubMed, Embase, Web of Science, Cochrane Library, Emcare, and Academic Search Premier were searched on August 16, 2019, to identify publications about digitization in the laboratory diagnostic setting (ie, web-based triage that leads to laboratory testing, self-sampling or testing, or web-based communication of laboratory test results). The search was updated on July 21, 2021. Search terms related to laboratory diagnostics and eHealth were combined (see Multimedia Appendix 1 for the full search strings). The search was limited to peer-reviewed publications. The reference lists of relevant reviews and the selected publications were also searched.

### Study Selection

The titles and abstracts of the identified publications were screened for relevance. The full text was screened when it concerned potentially relevant publications or when there was insufficient information in the abstract to adequately assess the relevance. Several inclusion criteria were used to select the relevant publications. First, the publication should focus on a *specific* web-based laboratory diagnostic service. The service could be (1) a web-based questionnaire or triage that directs users to a laboratory test (in the clinic or at home), (2) an ordered self-sampling or testing kit, or (3) a system for web-based communication of laboratory test results to users. Second, the laboratory diagnostic service should be (partly) independent of an HCP (eg, the questionnaire or triage should not be administered over the phone by the HCP; the test kit should not be provided in-person; administering the test should not require assistance from an HCP; and the test results should not be communicated through a phone call). Regarding the latter, the publication was included when it discussed a result service that was partly independent of an HCP (ie, negative test results were automatically communicated and, in case of positive test results, there was contact between the HCP and patient). Third, the publication should focus on primary care settings; however, this exclusion criterion was omitted for studies conducted in Africa (as there is no clear distinction between primary and secondary care). Fourth, the study outcomes should specifically examine the laboratory diagnostic service (ie, the triage, test, or web-based communication of the test results) and not the surrounding procedures (eg, the acceptability of the consent procedure or the development of the service). Relevant outcomes included actual use or uptake, feasibility and acceptability, and effectiveness (eg, the time taken to test for diagnosis, understanding of test results, and the accuracy of triage). Publications were excluded if the laboratory diagnostic service focused on (national) screening campaigns, the monitoring of disease progression, or retesting or increasing retesting rates. Reviews, trial protocols, non–peer-reviewed papers, non-English papers, and publications without data or with only hypothetical data were also excluded. AV screened all the titles, and AV and ET independently screened the abstracts and full-text publications. For the second search, which was used to update the data, KS screened all the titles. The screening of abstracts was performed independently by AV and KS, and full-text publication screening was performed independently by KS and ET. Discrepancies were resolved through discussion.

### Coding

A standardized coding form was used to extract all relevant information from the identified publications. The following information was extracted: (1) the first author and publication year, (2) the country in which the study was conducted, (3) the type of study design (using the classification by Hong et al [23]), and (4) sample characteristics (ie, target group, sample size, age, and gender). It was then determined which laboratory diagnostic service was studied (ie, web-based triage, self-sampling or testing, web-based result service, or any combination of the former three options). The names of the web-based laboratory diagnostic service and the recruitment method were also coded. The different recruitment methods were categorized as social marketing (eg, media, social media, magazines, flyers, advertisements, or promotion in target groups), community outreach (eg, face-to-face recruitment and community events), health service recruitment (ie, direct recruitment by the service provider in past service users), and other recruitment strategies. Details of the laboratory diagnostic services were extracted. Different data were collected based on what services or combinations of services were studied. For the web-based triage service, the aim of the triage was extracted, and it was determined whether it resulted in clinic- or home-based testing (ie, self-sampling or self-testing). For the self-sampling or self-testing service, the following information was extracted when applicable: (1) type of test (ie, self-sampling or self-testing); (2) for what disease; (3) type of specimen (eg, urine specimen); (4) method of how the test kit was ordered, delivered, and how the specimen could be returned; (5) method of instruction (ie, written or video); and (6) costs. For the web-based result service, we coded the method of result notification (eg, on the web or email), whether the notification was entirely or partially independent from an HCP, the average number of days before results were communicated, and whether individuals with positive results were linked to follow-up confirmatory testing or treatment. Results were then extracted, specifically results related to the service evaluation (see the *Study Selection* section) and not, for example, the characteristics of the service users. AV carried out the coding, and ET independently coded a subsample. There was substantial agreement between the 2 authors (ie, 77%). For the second search, the update, coding was done by KS.

### Quality Assessment

The quality of the included studies was assessed using the valid Mixed Method Appraisal Tool (MMAT) [23]. This tool was able to assess the quality of different study designs. The MMAT was chosen because it can be used to assess the methodological quality of 5 different study designs, specifically qualitative, randomized controlled, nonrandomized, quantitative descriptive, and mixed methods studies. The design was determined for each publication, and 5 corresponding quality criteria were rated. The criteria are shown in Multimedia Appendix 2. Each item was rated with *yes* (ie, indicative of good quality), *no* (ie, indicative of poor quality), or *can’t tell* (ie, insufficient evidence to determine the quality).

Furthermore, a numeric score was calculated to provide insight into the overall quality of each study. The AV conducted the complete quality assessment, and ET assessed a 10% subsample. The average Cohen κ was 0.80, indicating strong interrater reliability [24]. For the second search, KS completed the quality assessment of the studies (n=6).

### Data Analysis

Data were extracted from the results sections of the studies, as described in the coding paragraph. Relevant outcome measures were extracted verbatim and added to the database, enabling the clustering of different outcome measures. The main findings are presented separately for the different service types. A detailed description of the findings of the included studies is provided in Multimedia Appendix 3 [12-15,25-65].

## Results

### Study Selection

**Figure 1:**
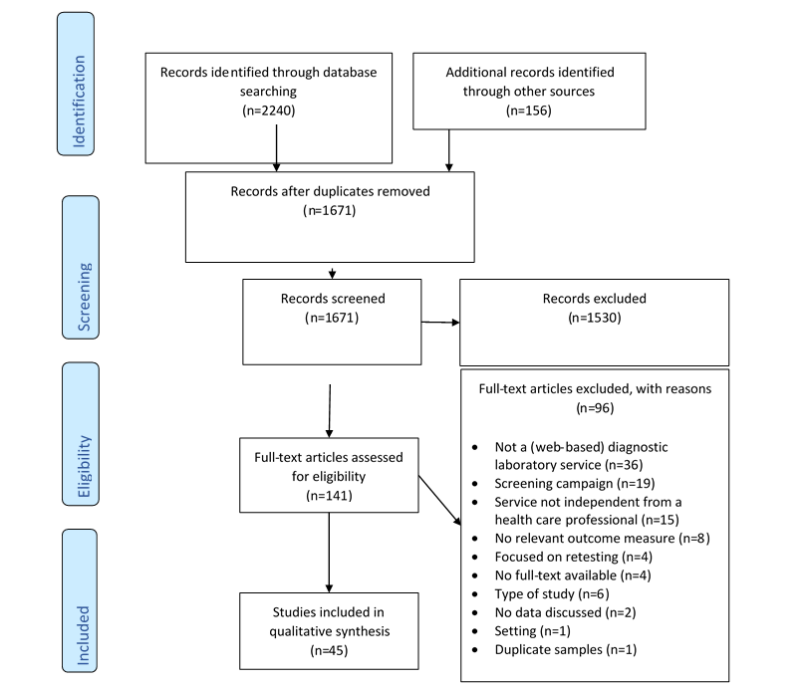
PRISMA (Preferred Reporting Item for Systematic Reviews and Meta-Analyses) flow diagram for study inclusion.

As shown in Figure 1, the 2 search strategies resulted in 1671 publications after removing duplicates. The titles and abstracts were screened for relevance, and the full texts of 141 publications were checked. A total of 96 publications were excluded, most frequently, because the publication did not report on a (web-based) diagnostic laboratory service (n=36), it concerned a national screening campaign (n=19), or the service was not independent of an HCP (n=15). Finally, 45 publications were included in the qualitative synthesis, and 6 studies were included in the second search.

### Study Characteristics

Most of the included studies had a quantitative descriptive design (n=28) [12,13,15,25-50]. In the remaining studies, a (quantitative) nonrandomized design was reported 6 times [32,51-55], a randomized controlled design was reported 5 times [56-60], a mixed methods design was reported 3 times [14,61,62], and a qualitative design was reported 3 times [63-65]. In 29 studies, a combination of services was offered; specifically, triage, testing, and a result service in 14 studies [13,28,40,42,46,49,51-53,56,57,59,60,63], triage and testing in 9 studies [26,27,29-33,35,37], and testing and a result service in 6 studies [41,44,45,48,61,64]. Furthermore, 8 studies discussed a testing service [14,25,34,38,43,47,58,62], 7 discussed a result service [15,35,39,50,54,55,65], and 1 discussed a triage service [12]. In the included studies, the testing service was evaluated most often (ie, 82% of the studies). Triage was evaluated in 2 studies [12,29] and the result service, in 11 studies [15,35,39-41,44,46,50,54,55,65]. The services were evaluated in the United States (n=15), the United Kingdom (n=9), Canada (n=6), Australia (n=2), Sweden (n=2), the Netherlands (n=2), and China (n=2). The remaining studies took place in Belgium, Brazil, Denmark, Estonia, France, Italy, and Uganda (ie, all n=1). The sample sizes ranged from 10 to 37 in the qualitative studies, with a mean of 21.60 (SD 9.7). The sample size ranged from 102 to 1736, with a mean of 2205.90 (SD 3514.0) in the quantitative studies. Almost half of the studies included both men and women (n=22) [12,13,25,29,36,38,39,48,50-57,59-62,64,65], 11 studies included MSM [27,28,34,35,41-43,45,47,49,63], 7 studies included only women [30-33,37,44,46], 2 studies included only men [26,58], 1 study included both MSM and transgender people [14], 1 study included adults with presumptive tuberculosis [15], and 1 study included past service users [40]. The mean percentage of male participants was 62.34% (SD 35.1%), and the mean age was 27.37 years (SD 4.7 years) (the average across the 15 studies that reported a mean) and ranged from 20.70 to 37.90 years. The study characteristics are shown in Table 1.

**Table 1:** Study characteristics.

Study and country	Study design	Study population	Sample size, n	Males, n (%)	Age (years)	Service type
Ahmed-Little et al, 2015 [61], the United Kingdom	Mixed methods	Persons aged ≥16 years	2247	1043 (46.41)	Mean 22.60	Testing^a^, result
Andersen et al, 2001 [25], Denmark	Quantitative descriptive	Persons aged 21 to 23 years	183	64 (34.9)	—^b^	Testing
Babirye et al, 2019 [15], Uganda	Quantitative descriptive	Adults with presumptive tuberculosis	233	114 (48.9)	IQR 27-50	Result
Barnard, 2018 [51], the United Kingdom	Quantitative nonrandomized	Personsaged ≥16 years	5747	2489 (43.31)	IQR 23-32	Triage, testing^a^, result
Brown, 2018 [56], the United Kingdom	Quantitative RCT^c^	High-risk persons aged ≥16 years	8999	7015 (77.95)	72% aged between 16 and 34	Triage, testing^a^, result
Chai, 2010 [26], the United States	Quantitative descriptive	Men aged ≥14 years	501	501 (100.00)	IQR 21-30	Triage, testing^a^
de Boni, 2019 [27], Brazil	Quantitative descriptive	MSM^d^ aged ≥18 years	3218	3218 (100.00)	IQR 22-31	Triage, testing^a^
Dulai, 2019 [49], Canada	Quantitative descriptive	Men who are gay, bisexual, and MSM aged ≥18 years	1272	1272 (100.00)	53% aged between 18 and 39	Triage, testing^a^, result
Elliot, 2016 [28], the United Kingdom	Quantitative descriptive	MSM	17,361	17,361 (100.00)	—	Triage, testing^a^, result
Grandahl et al, 2020 [64], Sweden	Qualitative	Personsaged ≥15 years	20	9 (45)	Mean 30.8	Testing^a^, result
Grandahl, 2020 [48], Sweden	Quantitative descriptive	Personsaged ≥15 years	1785	546 (30.58)	Mean 27.3	Testing^a^, result
Gaydos, 2016 [30], the United States	Quantitative descriptive	Women	102	0 (0)	64% aged between 18 and 29	Triage, testing^a^
Gaydos, 2016 [29], the United States	Quantitative descriptive	Persons aged ≥14 years	1394	558 (40.02)	Mean 28.13	Triage^a^, testing
Gaydos, 2011 [32], the United States	Quantitative nonrandomized	Women aged ≥14 years	1171	0 (0.00)	Mean 25.00	Triage, testing^a^
Gaydos, 2009 [31], the United States	Quantitative descriptive	Women aged ≥14 years	1203	0 (0.00)	Median 23	Triage, testing^a^
Gaydos, 2006 [33], the United States	Quantitative descriptive	Women aged ≥14 years	400	0 (0.00)	Mean 26.10	Triage, testing^a^
Gilbert, 2019 [52], Canada	Quantitative nonrandomized	Persons aged ≥14 years	381	270 (70.86)	Range 18-74	Triage, testing^a^, result^a^
Gilbert, 2017 [13], Canada	Quantitative descriptive	Persons aged ≥14 years	868	619 (71.31)	Median 32	Triage, testing^a^, result
Jin, 2019 [34], China	Quantitative descriptive	MSM aged ≥16 years	879	879 (100.00)	IQR 24-34	Testing
Kersaudy-Rahib, 2017 [57], France	Quantitative RCT	Persons aged 18-24 years	11,075	5152 (46.52)	Mean 20.70	Triage, testing^a^, result
Knight, 2018 [63], Canada	Qualitative	MSM aged ≥15 years	37	37 (100.00)	Mean 37.90	Triage, testing^a^, result
Koekenbier, 2008 [35], the Netherlands	Quantitative descriptive	MSM	898	898 (100.00)	—	Result
Kuder, 2015 [53], the United States	Quantitative nonrandomized	Persons aged ≥14 years	1211	484 (39.97)	Mean 27.47	Triage, testing^a^, result
Kwan, 2012 [36], Australia	Quantitative descriptive	Persons aged ≥16 years	377	206 (54.64)	71% were aged <30	Triage, testing^a^
Ladd, 2014 [37], the United States	Quantitative descriptive	Women	205	0 (0.00)	Mean 25.80	Triage, testing^a^
Ling, 2010 [54], the United States	Quantitative nonrandomized	Men and women	9056	5196 (57.37)	85% were aged ≥20	Result
Mák, 2015 [55], Canada	Quantitative nonrandomized	Persons aged ≥18 years	3292	1244 (37.79)	62% were aged ≥55	Result
Martin, 2009 [38], Australia	Quantitative descriptive	Persons aged 16-24 years	413	224 (54.2)	67% aged between 16 and 24	Testing
Morris, 2010 [39], the United States	Quantitative descriptive	Persons aged ≥18 years	3138	2563 (81.67)	62% aged between 25 and 44	Result
Nadarzynski, 2018 [40], the United Kingdom	Quantitative descriptive	Service users	115	—	—	Triage, testing, result^a^
Platteau, 2015 [41], Belgium	Quantitative descriptive	MSM aged ≥18 years	1071	1071 (100.00)	Mean 33.82	Testing, result^a^
Polilli, 2016 [12], Italy	Quantitative descriptive	Men and women	5000	—	—	Triage
Reagan, 2012 [58], the United States	Quantitative RCT	Men aged 18-45 years	200	200 (100.00)	Mean 30.75	Testing
Ricca, 2016 [42], the United States	Quantitative descriptive	MSM aged ≥18 years	896	896 (100.00)	Mean 30.00	Triage, testing^a^, result
Robinson, 2019 [65], Canada	Qualitative	No inclusion criteria	21	12 (57)	38% aged between 60 and 69	Result
Rosengren, 2016 [43], the United States	Quantitative descriptive	Black and Hispanic MSM aged ≥18 years	125	125 (100.00)	63% aged between 18 and 30	Testing
Rotblatt, 2013 [44], the United States	Quantitative descriptive	Women aged 12 to 25 years	2659	0 (0.00)	Median 22.3	Testing^a^, result^a^
Rüütel, 2015 [45], Estonia	Quantitative descriptive	MSM aged ≥18 years	265	265 (100.00)	53% were aged ≥30	Testing^a^, result
Spielberg, 2014 [46], the United States	Quantitative descriptive	Women aged 18-30 years	217	217 (100)	Median 25	Triage, testing^a^, result^a^
Talboom-Kamp, 2020 [50], the Netherlands	Quantitative descriptive	No inclusion criteria	354	—	—	Result
Wilson, 2019 [60], the United Kingdom	Quantitative RCT	Persons aged 16-30 years whom had never had a sexually transmitted infection test	528	254 (48.1)	Mean 21.30	Triage, testing^a^, result
Wilson, 2017 [59], the United Kingdom	Quantitative RCT	Persons aged 16-30 years	2063	846 (41.01)	Mean 23.00	Triage, testing^a^, result
Witzel, 2019 [14], the United Kingdom	Mixed methods	MSM and transgender people aged ≥16 years	1035/10	1035 (100)/ 10 (100)	IQR 26 to 42 or 60% aged between 26 and 40	Testing
Witzel, 2021[62], the United Kingdom	Mixed methods	Transgender people aged ≥16 years	118/20	94 (79.66)/ 12 (60)	IQR 22 to 37 or 35% aged between 16 and 25	Testing
Zhong, 2017 [47], China	Quantitative descriptive	MSM aged ≥18 years	380	380 (100)	54% aged between 25 and 34	Testing

^a^When multiple services were discussed in a study, footnote a identifies the service for which data was reported.

^b^—: data not available.

^c^RCT: randomized controlled trial.

^d^MSM: men who have sex with men.

### Service Provider Characteristics

Within the 45 studies included in this review, 31 different providers were examined. The characteristics of the service providers are shown in Table 2, and more details are provided in Appendix 4 [12-15,25-65]. About half of the service providers offered a combination of services. A total of 9 providers offered a triage, testing, and result service, 5 offered a testing and result service, and 2 offered a triage and testing service. The remaining providers offered a single service (ie, testing [n=7], result [n=7], or triage [n=1]). Social marketing was most often used to recruit service users or study participants, with 16 providers using it as the sole recruitment strategy and 5 providers combining it with community outreach. The health service recruited 7 providers, and 3 studies reported no information on the applied recruitment strategy.

Triage was offered by 12 different service providers, either alone or in combination with other services. Triage aimed to estimate the risk of having a disease and identify individuals who need to test. The aim of the triage, however, was not specified for 5 providers. In most cases, web-based triage directed users to home-based testing (83%). A total of 23 providers offered testing as a service (alone or in combination with other services); 12 providers offered testing for 1 disease, and 11 offered testing for >2 diseases (ie, ranging from 2 to 6). Testing was most often available for chlamydia (n=13), HIV (n=12), and gonorrhea (n=10). Providers also tested for trichomonas (n=3), syphilis (n=3), hepatitis B (n=1), hepatitis C (n=1), lymphogranuloma venereum (n=1), and mycoplasmosis (n=1). Most of the tests were performed with a self-sampling test (n=18), whereby the samples were returned to the laboratory and analyzed according to the gold standard. All laboratories provided high-quality analysis with accredited and certified equipment. Self-testing was offered by 5 providers and targeted HIV (n=5) and syphilis (n=1). The testing service was almost always free of charge (87%). A small shipping fee was charged by 1 provider, and 1 provider charged US $23 that would be refunded after the user had shared the test results with the staff. A result service was offered by 20 providers (alone or in combination with other services). Different methods were used to communicate the test results, with 8 providers relying on a single method and 10 providers using different methods for result communication. Test results were most often accessible on the internet (n=12) or communicated over the phone (n=10). The results could also be communicated using SMS text messaging (n=6) or email (n=2). The communication of the test results was, in most cases, not completely independent from an HCP (70%). Often, the results were presented on the web, but users were called by the HCP when they had a positive result [39,63], or users were called when they had not checked their results on the internet [41].

**Table 2:** A description of the diagnostic testing and result service provider.

Service provider		Recruitment method^a^	Triage, type of follow-up testing	Testing				Result	
				Diseases	Type of home-based test	Cost on average (US $)	Method		Independent health care provider
**Triage service**									
	Fai il test anche TU project [12]	Social	Clinic	HIV, hepatitis B and C, syphilis	—^b^	—	—		—
**Testing service**									
	C-project [38]	Social	—	Chlamydia	Self-sampling	Free	—		—
	Easy test [34]	Social; Community	—	HIV	Self-testing	2-3	—		—
	UCLA free HIV self-test program [43]	Social	—	HIV	Self-testing	Free	—		—
	Social entrepreneurship testing [47]	—	—	HIV, syphilis	Self-testing	23 (refunded)	—		—
	SELPHI [14,62]	Social	—	HIV	Self-testing	Free	—		—
	Unknown [25]	Social	—	Chlamydia	Self-sampling	Free	—		—
	Unknown [58]	Social; Community	—	Chlamydia, gonorrhea	Self-sampling	Free	—		—
	Unknown [48,64]	Health service	—	Chlamydia, gonorrhea	Self-sampling	Free	—		—
**Result service**									
	GxAlert [15]	Health service	—	Tuberculosis	—	—	SMS text messaging		Yes
	Syfilistest.nl [35]	Social	—	Syphilis	—	—	Web-based		Yes
	Early test [39]	Social	—	HIV	—	—	Web-based; phone		Partly
	Result system of Denver Metro Health Clinic [54]	Health service	—	Chlamydia, gonorrhea	—	—	Web-based		Partly
	Excelleris [55]	Health service	—	Not limited to a specific disease	—	—	Web-based		Yes
	Patient portal [50]	Health service	—	Not limited to a specific disease	—	—	Web-based		Partly
	myCARE [65]	Health service	—	Not limited to a specific disease	—	—	Web-based		Partly
**Triage and testing service**									
	A hora é Agora [27]	Social	Home	HIV	Self-testing	Free	—		—
	Online Chlamydia Testing program [36]	Social	Home	Chlamydia, gonorrhea	Self-sampling	Free	—		—
**Testing and result service**									
	Swab2Know [41]	Social	—	HIV	Self-sampling	Free	Web-based; email; phone		Partly
	Do not think, know [44]	Social; Community	—	Chlamydia, gonorrhea	Self-sampling	Free	Web-based; phone		Partly
	Testikodus [45]	Social	—	Chlamydia, gonorrhea, trichomonas, LGV^c^, mycoplasmosis	Self-sampling	Free	Web-based		Yes
	RUClear [61]	—	—	HIV	Self-sampling	—	Phone; SMS text messaging; letter		Partly
**Triage, testing, and result service**									
	DS@H [28]	Social	Home	HIV	Self-sampling	Free	SMS text messaging; web-based; phone		Partly
	GetCheckedOnline [13,49,52,63]^d^	Social	Home, clinic	Chlamydia, gonorrhea	Self-sampling	Free	Web-based; phone		Partly
	Let’s talk about it NHS [40]	Health service	Home	Chlamydia, gonorrhea, HIV, syphilis, hepatitis B and C	Self-sampling	Free	SMS text messaging; phone		Partly
	Checking in [42]	Social	Home	HIV	Self-sampling	Free	Phone		Partly
	eSTI [46]	Social; Community	Home	Chlamydia, gonorrhea, trichomonas	Self-sampling	Free	Web-based		Yes
	SH:24 [48,59,60]^d^	Social; Community	Home	Chlamydia, gonorrhea, HIV, syphilis	Self-sampling	Free	SMS text messaging; phone		Partly
	Freetesting.hiv [56]	—	Home	HIV	Self-sampling	Free	SMS text messaging; phone		Partly
	Chlamyweb [57]	Social	Home	Chlamydia	Self-sampling	Free	Email; postal service		Partly
	I Want The Kit [26,29-33,37,53]^d^	Social	Home	Chlamydia, gonorrhea, trichomonas	Self-sampling	Free	Web-based		Yes

^a^The methods used to recruit participants or service users was reported; specifically, social=social marketing, community=community outreach, and health service=health service recruitment.

^b^Data not available.

^c^Lymfogranuloma venereum.

^d^The service provider was investigated in multiple studies. The specific characteristics of each study are presented in Multimedia Appendix 3.

### Quality Assessment

Quality assessment using the MMAT of the studies is shown in Table 3. The quality of the included studies was good, with an average score of 3.86 (SD 0.6; on a scale from 0 to 6). The average quality score ranged from 3.33 (SD 1.5) for mixed methods studies to 4.67 (SD 0.57) for qualitative studies. A shortcoming was that, in the studies using a quantitative descriptive design, the nonresponse was not clearly reported in 23 of the 25 studies. Therefore, it is unclear if these studies were at risk of nonresponse bias.

**Table 3:** Quality assessment of the included studies using the Mixed Method Appraisal Tool (MMAT).

Included studies		MMAT quality criteria^a^						MMAT scores^b^	
		1	2	3	4	5			
**Qualitative**									4.67
	Knight et al [63]	+^c^	+	+	+	+	5		
	Grandahl et al [64]	+	+	+	+	+	5		
	Robinson et al [65]	(+/−)^d^	+	+	+	+	4		
**Quantitative randomized controlled trials**									4.20
	Brown et al [56]	+	+	+	+/−	+	4		
	Kersaudy-Rahib et al [57]	+	+	−^e^	+/−	+	3		
	Reagan et al [58]	+	+	−	+	+	4		
	Wilson et al [59]	+	+	+	+	+	5		
	Wilson et al [60]	+	+	+	+	+	5		
**Quantitative nonrandomized**									3.83
	Gaydos et al [32]	+	+	+	+/−	+	4		
	Barnard et al [51]	+	+	−	+	+	4		
	Gilbert et al [52]	−	+	+/−	+	+	3		
	Kuder et al [53]	+	+	−	−	+	3		
	Ling et al [54]	+	+	+	+	+	5		
	Mák et al [55]	−	+	+	+	+	4		
**Quantitative descriptive**									3.78
	Polilli et al [12]	+	+	+	+/−	+	4		
	Gilbert et al [13]	+	+	+	+/−	+	4		
	Babirye et al [15]	+	+	+	+	+	5		
	Andersen et al [25]	+	+	+	+/−	+	4		
	Chai et al [26]	+	+	+	+/−	+	4		
	de Boni et al [27]	+	+	+	+/−	+	4		
	Elliot et al [28]	+	+	+	+/−	+/−	3		
	Gaydos et al [29]	+	+	+	+/−	+	4		
	Gaydos et al [30]	+	+	+	+/−	+	4		
	Gaydos et al [31]	+	+	+	+/−	+	4		
	Gaydos et al [33]	+	+	+	+/−	+	4		
	Jin et al [34]	+	+	+	+/−	+	4		
	Koekenbier et al [35]	+	+	+	+/−	+	4		
	Kwan et al [36]	+	−	+	+/−	+	3		
	Ladd et al [37]	+	+	+	+/−	+	4		
	Martin et al [38]	+	−	+	+/−	+	3		
	Morris et al [39]	+	+	+	+/−	+	4		
	Nadarzynski et al [40]	+	+/−	+	+/−	+	3		
	Platteau et al [41]	+	+	−	+/−	+	3		
	Ricca et al [42]	+	+	+	+/−	+	4		
	Rosengren et al [43]	+	+	+	+/−	+	4		
	Rotblatt et al [44]	+	+	+	+/−	+	4		
	Rüütel et al [45]	+	−	+	−	+	3		
	Spielberg et al [46]	+	+	+	+/−	+	4		
	Zhong et al [47]	+/−	+	+	+/−	+	3		
	Grandahl et al [48]	+	+	+	−	+	4		
	Dulai et al [49]	+	+	+	−	+	4		
	Talboom-Kamp et al [50]	+	+	+	−	+	4		
**Mixed methods**									3.33
	Witzel et al [14]	+	+	+	+	−	4		
	Ahmed-Little et al [61]	+/−	−	+	+	−	2		
	Witzel et al [62]	+	+	+	+/−	+	4		

^a^The criteria differed according to the design. A description of the criteria is provided in Multimedia Appendix 2.

^b^The average Mixed Method Appraisal Tool score across all designs is 3.86. The overall grade is the sum of the number of quality criteria that were assessed as good.

^c^Good quality.

^d^Insufficient evidence to determine the quality.

^e^Poor quality.

### Findings by Type of Service

#### Overview

The findings are discussed separately for triage, testing, and result service. For clarity, the findings of follow-up testing and treatment are jointly discussed for the testing and result service. A more detailed description of the findings is provided in Multimedia Appendix 4.

#### Triage Service

A total of 2 studies evaluated the triage service, which showed that the use of web-based triage services could be quite high with those completing the web-based triage and booking an appointment for a test (more than 50%). Notably, most of the individuals who tested positive were also linked to treatment. Furthermore, the predictive value of triage showed a prediction of STI positivity in women. For more detailed information, see Table 4.

**Table 4:** Results of the triage and test services per specific outcome measure.

Service and general outcome		Specific outcome measure	Results
**Triage**			
	**—^a^**		
		Use	Use of web-based triage services can be quite high; more than 50% (3046/5000) of those who completed the web-based triage also booked an appointment for HIV clinic-based testing. Notably, the majority also presented for testing (87%), and most of the individuals who tested positive were also linked to treatment (93%) [12]
		Predictive value	Gaydos et al [29] found that the score on the risk assessment predicted STI^b^ positivity for females but not males
**Test**			
	**Use**		
		Return specimen	The percentage of returned tests or specimens for analyses was frequently reported [13,25,26,28,37,38,42,44-46,48,51,56,61] Range: 24 [45] to 85% [42,48]; mean 52.8% (SD 19.6%)
		Used tests	In 4 studies, the percentage of used home-based tests was given [14,36,43,47] Range: 56 [36] to 100% [43]; mean 83% (SD 19.3%) The highest percentage might be an overestimation of the actual use because people had to self-report the use of the tests in a follow-up survey [43]
		Comparison home-based testing vs clinic-based testing	In 4 studies, home-based testing was compared with clinic testing [57-60] The average percentage of test use was higher among those who were offered a home test compared with those who were offered a test at the clinic (mean 49%, SD 17.8% vs mean 27%, SD 16.1%, respectively)
		Other	Home-based test uptake was highest when the results would be presented through the internet [53] When users received primers before the arrival of the test kit at home (eg, set aside a time to complete the test) and behavioral insight reminders [56]
	**Acceptability or usability**		
		Home-based testing vs clinic-based testing	Eight studies examined whether there was a preference for home-based or clinic-based testing [26,30,32,33,43,46,63] Range: 62 [30] to 95% [46]; mean 81% (SD 12.7%) who preferred home-based testing One study reported a barrier to clinic-based testing: that it was easier to stay at home than go to the clinic [49]
		Easy to perform	Seven studies reported how easy it was to perform home-based testing [14,26,30,32,33,36,43] Range: 88% [26] to 97% [14,32]; mean 94% (SD 3.5%)
		Acceptability instructions	Five studies examined the acceptability of the instructions for home-based testing [14,27,30,58,61] Mean 93% (SD 5.3%) considered the instructions to be easy.
		Acceptability in general	In 3 studies, the acceptability of the home-based test service, in general, was reported [59-61] Mean 75% (SD 4.5%)
		Recommendation	The percentage of participants who would recommend the service of testing at home to a friend was 98% in 2 studies [36,46], and in Gaydos et al [30], it was 77%
		Other	The perceived reliability of the test results was reported in Gaydos et al [30]: 97% of the users trusted the results of the home-based test service Chai et al [26] found that 85% found it a safe way of testing Witzel et al [14] found that 97% had an overall good experience with the home-based test service Chai et al [26], Gaydos et al [32], and Dulai et al [49] both reported that around 90% would use the home-based test service again Gaydos et al [33] report that 86% would use this home-based testing method in daily life de Boni et al [27] reported that 91% found it (very) easy to use the website Grandahl et al [48] reported that more than 90% found the overall home-based test service good or very good Grandahl et al [64] reported that most users highly appreciated the service and found the service easy to use, convenient, and confidential. They would use the service again in the future, even if the costs were higher
	**Cost-effectiveness**	Cost-effectiveness	Kersaudy-Rahib et al [57] reported that the price for home-based testing was three times lower compared with clinic-based testing Ahmed-Little et al [61] showed that the costs for HIV testing per person were around “€27 (US $ 30.45), which is in line with testing costs in national HIV testing pilots
	**Other outcomes**	—	The reasons to self-test were that it reduced HIV testing barriers, desire to use new technology, and altruistic motivation [14] Other reasons mentioned for HIV self-testing were inaccessible and inappropriate clinical services [62]. In Martin et al [38] users reported that they did the test because it was easy and it was for free Zhong et al [47] reported convenience and to save time, protection of privacy, ease of use, and accuracy as reasons to perform a home-based self-test. Facilitators were ease of use, anonymity, and the ability to test alone. Barriers were concerns about accuracy, potential costs, and concerns about self-interpreting the results Dulai et al [49] reported that 20% were worried about their online information privacy, and 5% had low trust in this service Some barriers mentioned in Grandahl et al [64] were the use of complicated language, uncertainty about the procedure, unreliable postal service, and insecure data handling

^a^No general outcome measure.

^b^STI: sexually transmitted infection.

#### Testing Service

For the test service, different outcome measures were found with different objectives. Studies with outcomes focusing on the test services, which were home-based (eg, self-testing or self-sampling), were discussed. The test use was reported to be high (above 50%), and test uptake was higher among those offered home-based tests than clinic-based tests. The number of returned specimens was discussed frequently and showed very different results with a wide range of percentages of returned specimens. The acceptability and usability of the test service scored high on the convenience of performing home-based tests with easy instructions. The cost-effectiveness of home-based tests showed lower or similar prices compared with clinic-based testing. Furthermore, motivations for self-testing were discussed. Ease of use, privacy, and anonymity were identified as reasons to perform these tests. Important barriers for these services were potential costs, accuracy, unreliable postal service, insecurity about handling data, and self-interpreting the results. For more detailed information, see Table 4.

#### Result Service

For the result service, different types of outcome measures were found with different objectives. The use of the result service exceeded 69%. Research showed that most participants viewed their results on the same day as they were posted on the web, and comprehension of these web-based results was high (above 75%). The acceptability of direct access to results using the website was high, and the participants were satisfied with this process. Direct access to diagnostic results led to shorter waiting times for the results than for participants who did not receive their results on the web. Limited access to the internet was a reason for preferring to call the clinic for the results. For more detailed information, see Table 5.

**Table 5:** Results of the test and result services per specific outcome measure.

Service and general outcome		Specific outcome measure	Results
**Result**			
	**Use**		
		Retrieved results on the internet	The use of a result service was assessed in 6 studies [35,39,41,44,46,54] The percentage of people who retrieved their results on the internet varied from 69 [39] to 97% [35]; mean 85% (SD 11.2%) The service with the lowest retrieval rate called all users with a positive test result and, if users were not called within 2 week they could access their results on the internet Spielberg et al [46] found that 88% viewed their test results on the same day that the results were posted Platteau et al [41] showed that significantly more people collected their test results when the test was ordered online compared with testing during outreach activities
		Waiting time	Gilbert et al [52] showed significantly shorter waiting times for those who used a web-based platform compared with clinic clients
	**Comprehension**	—^a^	Babirye et al [15] found that everyone could accurately relay the content of an SMS text message that contained the tuberculosis test result Comprehension was slightly lower in the other 2 studies: 75% and 87% understood the content of the test result message, respectively [40,55] Mák et al [55] showed that comprehension was significantly higher in the group that did not receive their results on the internet Robinson et al [65] showed that comprehension of the results differed from difficulty with the understanding of the results to no difficulty. However, when difficulties were there, the users pointed out that the reference range was helpful.
	**Acceptability**		
		Comfortable with web-based results	The acceptability was examined in 4 different studies [39,41,46,54] Only 1 study specifically examined how comfortable users were with receiving their results on the internet, and 87% was (very) comfortable with this process [39]
		Ordering a test and receiving results on the web	Two studies examined the acceptability of ordering a test kit on the web and receiving the web-based results Platteau et al [41] found that 96% of the users were satisfied with this process Spielberg et al [46] reported that 98% of the users found the service website easy to use
		Reasons	The two main reasons for choosing to receive web-based results were having access to the results any time of the day and the belief that results would be communicated faster via the internet A preference to call the clinic for results and limited access to the internet were reasons to opt-out of web-based results [54] The reasons for using web-based results were reported by Robinson et al [65] as better communication with the HCP^b^, convenience, and being a steward of your health care
	**Other outcomes**	—	The feasibility of using SMS text message to communicate tuberculosis test results was examined in Uganda and scored relatively low; (ie, an SMS text message was only transmitted to 62% of those who were eligible to receive an SMS text message with test results [15]) One study found that users waited significantly shorter for web-based test results than users who did not have web-based access [55]. Furthermore, this study showed that the majority (ie, 86%) experienced no or low anxiety after receiving their test results, and the level of anxiety was not different between those with or without internet access Another study examined user preferences for the content of the text messages conveying the test results, and the majority preferred that the results of all tested STIs^c^ were discussed in one message and that the names of the STIs tested should be included in the message [40] One study reported that patients feel more comfortable and engaged with their health care when they see the results themselves [65]. Besides, they reported that it had no adverse effects Two domains of the eHIQ^d^ were researched in one study to determine patient’s attitude toward a web-based results service [50]. This eHIQ showed positive results for the criteria: easy to use, trustworthy, and appropriate
**Test and result**			
	**Follow-up testing and treatment**		
		Confirmatory testing	The frequency of confirmatory testing for positive or uncertain or invalid test results was described in 4 studies [27,35,43,61] Range from 68% [27] to 100% [43,61]; mean 85% (SD 17.7%)
		Follow-up after positive result	Follow-up treatment after a positive test result was described in 10 studies [26,31,32,34,36,41-44,46] Receiving web-based test results led to high treatment rates; mean 93% (SD 9.9%)
		Confirmatory testing and treatment	In 2 studies, confirmatory testing and treatment were described [28,47] In Elliot et al [28], 67% of the reactive samples were confirmed, and all received treatment. For 10% of the reactive samples, treatment could not be confirmed In Zhong et al [47], everyone with a reactive test did confirmatory testing and was linked to treatment
		Other	In 3 studies, different groups were compared with each other. It was shown that the treatment rate was higher when users (1) had the option to receive web-based results versus communicated over the phone (not significant) [54], (2) received their test kit at home instead of at the primary care setting [57], and (3) received their results through an automated result access system compared with service where participants had to call for their test result [53]

^a^Data not available.

^b^HCP: health care professional.

^c^STI: sexually transmitted infection.

^d^eHIQ: e-Health Impact Questionnaire.

### Test and Result Services: Follow-Up Testing and Treatment

Follow-up testing and treatment have been discussed in several studies. These studies showed that receiving web-based results led to high treatment rates (mean 93%, SD 9.9%), and the frequency of confirmatory testing after a self-test was above 68%. For more details, see Table 5.

## Discussion

### Principal Findings

This systematic review aimed to gain insight into the available methods for direct web-based access to patients for diagnostic testing and results. A total of 45 studies were included. Most of the studies used a quantitative descriptive design. Most of the studies investigated a test or result service related to STIs. In the 45 studies, 31 different providers were discussed. Half of the providers offered a combination of services. Of the 3 different services, the test service was most often evaluated. This review showed that direct patient access to testing and result services was positively evaluated. The use of triage, test, and result services was high, and the acceptability among patients was high. Moreover, follow-up confirmatory testing and treatment rates were high with home-based testing.

An update of the literature search was performed after the third wave of the COVID-19 pandemic. However, no studies were found regarding direct access to diagnostic testing and results services for this disease. This could be because free tests were often offered by the governments of countries. There have been commercial companies offering tests for SARS-CoV-2; however, scientific research has not yet been performed.

This review found that the use rates of home-based tests were high and that direct web-based access to results was appreciated and generally well-understood. An overall preference for home-based testing versus clinic-based testing was found. Importantly, follow-up treatment after a positive home-based test was high and, in some studies, was even higher when tests were performed at home compared with the clinic. The overall positive findings of this systematic review contradict earlier voiced concerns about self-testing and self-sampling, such as that users would be insufficiently linked to follow-up testing or treatment [66,67]. It was reported in 1 study that 70% of participants were afraid to carry out the self-test properly [67]. This contrasted with our findings, which indicated that users found self-tests easy to use and that the instructions were clear and reliable. Nevertheless, it is important to include end users in the design phase when setting up such services to ensure usability and acceptability [68]. In addition, although most studies reported high acceptability and comprehension of test results communicated on the web, 1 study reported that interpreting the results was easier when they were communicated in person (vs via the internet). This contradictory finding might be because this study discussed a general result service portal and not a portal specifically for STI results. To minimize the risk of misunderstanding, it is important that future research examine the content and how this content can best be presented to users [50].

Furthermore, the quality of the laboratory tests used in these studies was high. Therefore, this review disproves the aforementioned concerns about home-based diagnostic tests [66,67] and shows that these tests with direct access to web-based result services could contribute to easily accessible diagnostic testing [69].

The high acceptability of the test and result services and the high rates of follow-up for treatment create opportunities for primary care. The workload for primary care is high [3,4]. eHealth technologies can make health care delivery more efficient, and therefore, the adoption of eHealth is being stimulated worldwide [9]. By providing patients with direct access to web-based testing and results, patients would not need to visit their HCP, potentially lowering the number of consultations in primary care. Consequently, it would leave HCPs with more time to focus on complex health care and consultations that cannot be executed via the internet. Another reason for home-based diagnostic testing is to lower the testing threshold. Patients can experience feelings of embarrassment or shame for tests such as STI, which can result in delays in testing [70]. Allowing individuals to order tests on the web can make it more convenient for them to get tested and may help diagnose and treat diseases sooner. However, future research should investigate whether these types of test services lead to excessive use. At the same time, it is important to emphasize that this review identified that direct access to diagnostic testing exhibited benefits for patients, such as comfort, ease, and time-saving. A few barriers should be addressed to allow home-based diagnostic testing in practice. An important barrier to eHealth adoption in primary care is, for example, the cost [71]. In the Netherlands, diagnostic tests ordered by a primary care physician are covered by health insurance. However, home-based diagnostic testing has not yet been covered by insurance. To stimulate home-based testing, the costs of home-based diagnostic testing should be covered by an individual’s health care insurance. Therefore, it would be useful to investigate the cost-effectiveness of home-based diagnostic testing compared with clinic-based testing. In this review, only 2 studies discussed cost-effectiveness, more insight into how valuable home-based diagnostic testing could be in the future could be provided. Furthermore, home-based diagnostic testing could work more efficiently in primary care if implemented for a variety of conditions [72]. However, more research is needed to elaborate on home-based diagnostic test services for diseases other than STIs.

### Strengths and Limitations

The strengths of this review lie in several aspects. First, the study search strategy was comprehensive and not limited to a specific disease or population. Second, a quality assessment was performed for all included studies, and the quality of the included studies appeared to be relatively high. However, it is essential to consider that the MMAT was scored using a yes or no score without nuances. Third, a comprehensive overview of the study and service characteristics provided detailed insight into the included studies.

This review has several limitations. First, there was heterogeneity in the included outcome measures, which resulted in a low number of studies reporting the same outcome. Therefore, it was not possible to examine the pooled effect using a meta-analysis. As the field advances quickly, more studies are likely to become available soon, and a meta-analysis might be possible. Second, almost all studies focused on STIs. For that reason, it was unknown whether the findings regarding usability and acceptability would generalize to test and result services that target diseases other than STIs. Nevertheless, our review provided insight into the potential of direct web-based access to diagnostic testing, which could translate to other diseases. Even for test results that were not dichotomous, which was the case in STI testing, test results could be presented in a web-based portal, for example, the identification of abnormal and normal values for a test result with an option to contact a physician [50]. A third limitation was that the mean age in the included studies was relatively low, which could have led to bias because a different, older population could have evaluated these services differently [73]. Although eHealth services have shown good use and result in older adult populations, it remains to be determined whether this is also the case for web-based diagnostic testing and results services [74]. There was a large portion of the quantitative descriptive design studies (28/45, 62%) that constituted the fourth limitation to this review. Only 5 studies had a randomized controlled trial design. Therefore, selection bias cannot be ruled out, including sample representativeness. Nevertheless, all studies underwent quality assessment and scored relatively high.

### Conclusions

Home-based testing showed higher use rates and follow-up treatment rates compared with clinic-based testing. It was demonstrated to be acceptable, safe, and convenient for users, which could lower the threshold for testing. Future research on diagnostic testing for diseases other than STIs and cost-effectiveness evaluation is needed. To conclude, this review showed that eHealth technologies for diagnostic testing could contribute to easy direct access to high-quality diagnostic testing for patients and has the potential to increase efficiency and possibility to reduce workload in primary care. In conclusion, direct web-based access to diagnostic testing showed promising results.

## Appendix

Multimedia Appendix 1 Search terms for this systematic review.

Multimedia Appendix 2 Mixed Method Appraisal Tool.

Multimedia Appendix 3 Server provider characteristics per study.

Multimedia Appendix 4 Overview of the reported outcomes for the triage, testing, and result services.

## Abbreviations

HCP: health care professional
MMAT: Mixed Method Appraisal Tool
MSM: men who have sex with men
PRISMA: Preferred Reporting Items for Systematic Reviews and Meta-Analyses
STI: sexually transmitted infection
