# Supplementary material for: Direct Access for Patients to Diagnostic Testing and Results Using eHealth: Systematic Review on eHealth and Diagnostics
Source: J Med Internet Res. 2022 Jan 12;24(1):e29303. doi: 10.2196/29303 (PMC8792777; doi:10.2196/29303)
Supplement: Multimedia Appendix 1 [file jmir_v24i1e29303_app1.docx]

**Appendix 1.** Search terms for this systematic review.

**PubMed**

http://www.ncbi.nlm.nih.gov/pubmed?otool=leiden

**(**(("Clinical Laboratory Techniques"[majr:noexp] OR "Laboratory Technique"[tw] OR "Laboratory Techniques"[tw] OR "laboratory diagnosis"[tw] OR "Clinical Laboratory Tests"[tw] OR "Laboratory Test"[tw] OR "Laboratory Testing"[tw] OR "lab test"[tw] OR "lab tests"[tw] OR "lab testing"[tw] OR "Laboratory Examination"[tw] OR "diagnostic tool"[tw] OR diagnostic tool*[tw] OR "diagnostic assessment"[tw] OR diagnostic assessment*[tw] OR "diagnostic system"[tw] OR diagnostic system*[tw] OR "diagnostic test"[tw] OR diagnostic test*[tw] OR "self-test"[tw] OR self test*[tw] OR "home-based test"[tw] OR home-based test*[tw] OR "self-sampling"[tw] OR postal test*[tw] OR test kit*[tw] OR testing kit*[tw] OR tests kit*[tw] OR STI test*[tw] OR STD test*[tw] OR testing program*[tw] OR "HIVST"[tw] OR "self-swabbing"[tw]) AND ("health information technology"[ti] OR "health information systems"[ti] OR "interactive health communication"[ti] OR "patient portal"[ti] OR "Telemedicine"[majr] OR web portal*[ti] OR telemed*[ti] OR "ehealth"[ti] OR "e-health"[ti] OR "mhealth"[ti] OR "m-health"[ti] OR "mobile health"[ti] OR "telehealth"[ti] OR "tele-health"[ti] OR "tele health"[ti] OR "webbased"[ti] OR "web-based"[ti] OR "telemedicine"[ti] OR "tele-care"[ti] OR "telecare"[ti] OR "website"[ti] OR "websites"[ti] OR "webpage"[ti] OR "webpages"[ti] OR "web application"[ti] OR "web applications"[ti] OR "web access"[ti] OR "Internet"[majr] OR "internet"[ti] OR "online communication"[ti] OR "on-line communication"[ti] OR "on line communication"[ti] OR text message*[ti] OR "sms"[ti] OR "smart message service"[ti] OR "short message service"[ti]) NOT ("Animals"[mesh] NOT "Humans"[mesh])) **OR** "DirectLab"[all fields] OR "swab2know"[all fields] OR "getcheckedonline"[all fields] OR "e-STI"[all fields] OR "WeTest"[all fields] OR "SELPHI"[all fields] OR "eSexual"[all fields] OR "chlamyweb"[all fields]**)**

**Embase**

http://ovidsp.ovid.com/ovidweb.cgi?T=JS&PAGE=main&MODE=ovid&D=oemezd

**(**(("Laboratory Technique".ti,ab OR "Laboratory Techniques".ti,ab OR exp *"laboratory diagnosis"/ OR "laboratory diagnosis".ti,ab OR "Clinical Laboratory Tests".ti,ab OR "Laboratory Test".ti,ab OR "Laboratory Testing".ti,ab OR "lab test".ti,ab OR "lab tests".ti,ab OR "lab testing".ti,ab OR "Laboratory Examination".ti,ab OR "diagnostic tool".ti,ab OR diagnostic tool*.ti,ab OR "diagnostic assessment".ti,ab OR diagnostic assessment*.ti,ab OR "diagnostic system".ti,ab OR diagnostic system*.ti,ab OR exp *"diagnostic test"/ OR "diagnostic test".ti,ab OR diagnostic test*.ti,ab OR "self-test".ti,ab OR self test*.ti,ab OR "home-based test".ti,ab OR home-based test*.ti,ab OR "self-sampling".ti,ab OR postal test*.ti,ab OR test kit*.ti,ab OR testing kit*.ti,ab OR tests kit*.ti,ab OR STI test*.ti,ab OR STD test*.ti,ab OR testing program*.ti,ab OR "HIVST".ti,ab OR "self-swabbing".ti,ab) AND ("health information technology".ti OR "health information systems".ti OR "interactive health communication".ti OR "patient portal".ti OR exp *"Telemedicine"/ OR exp *"Telehealth"/ OR "web portal*".ti OR telemed*.ti OR "ehealth".ti OR "e-health".ti OR "mhealth".ti OR "m-health".ti OR "mobile health".ti OR "telehealth".ti OR "tele-health".ti OR "tele health".ti OR "webbased".ti OR "web-based".ti OR "telemedicine".ti OR "tele-care".ti OR "telecare".ti OR "website".ti OR "websites".ti OR "webpage".ti OR "webpages".ti OR "web application".ti OR "web applications".ti OR "web access".ti OR exp *"Internet"/ OR "internet".ti OR "online communication".ti OR "on-line communication".ti OR "on line communication".ti OR text message*.ti OR "sms".ti OR "smart message service".ti OR "short message service".ti) NOT (exp "Animals"/ NOT exp "Humans"/)) **OR** "DirectLab".af OR "swab2know".af OR "getcheckedonline".af OR "e-STI".af OR "WeTest".af OR "SELPHI".af OR "eSexual".af OR "chlamyweb".af**)**

NOT (conference review or conference abstract).pt

**Web of Science**

http://isiknowledge.com/wos

**(**(TS=("Laboratory Technique" OR "Laboratory Techniques" OR "laboratory diagnosis" OR "laboratory diagnosis" OR "Clinical Laboratory Tests" OR "Laboratory Test" OR "Laboratory Testing" OR "lab test" OR "lab tests" OR "lab testing" OR "Laboratory Examination" OR "diagnostic tool" OR "diagnostic tool*" OR "diagnostic assessment" OR "diagnostic assessment*" OR "diagnostic system" OR "diagnostic system*" OR "diagnostic test" OR "diagnostic test" OR "diagnostic test*" OR "self-test" OR "self test*" OR "home-based test" OR "home-based test*" OR "self-sampling" OR "postal test*" OR "test kit*" OR "testing kit*" OR "tests kit*" OR "STI test*" OR "STD test*" OR "testing program*" OR "HIVST" OR "self-swabbing") AND TI=("health information technology" OR "health information systems" OR "interactive health communication" OR "patient portal" OR "Telemedicine" OR "Telehealth" OR "web portal*" OR telemed* OR "ehealth" OR "e-health" OR "mhealth" OR "m-health" OR "mobile health" OR "telehealth" OR "tele-health" OR "tele health" OR "webbased" OR "web-based" OR "telemedicine" OR "tele-care" OR "telecare" OR "website" OR "websites" OR "webpage" OR "webpages" OR "web application" OR "web applications" OR "web access" OR "Internet" OR "internet" OR "online communication" OR "on-line communication" OR "on line communication" OR "text message*" OR "sms" OR "smart message service" OR "short message service") NOT ti=("veterinary" OR "rabbit" OR "rabbits" OR "animal" OR "animals" OR "mouse" OR "mice" OR "rodent" OR "rodents" OR "rat" OR "rats" OR "pig" OR "pigs" OR "porcine" OR "horse" OR "horses" OR "equine" OR "cow" OR "cows" OR "bovine" OR "goat" OR "goats" OR "sheep" OR "ovine" OR "canine" OR "dog" OR "dogs" OR "feline" OR "cat" OR "cats")) **OR** ts=("DirectLab" OR "swab2know" OR "getcheckedonline" OR "e-STI" OR "WeTest" OR "SELPHI" OR "eSexual" OR "chlamyweb")**)**

**Cochrane Library**

https://www.cochranelibrary.com/advanced-search/search-manager

**(**(("Laboratory Technique" OR "Laboratory Techniques" OR "laboratory diagnosis" OR "laboratory diagnosis" OR "Clinical Laboratory Tests" OR "Laboratory Test" OR "Laboratory Testing" OR "lab test" OR "lab tests" OR "lab testing" OR "Laboratory Examination" OR "diagnostic tool" OR "diagnostic tool*" OR "diagnostic assessment" OR "diagnostic assessment*" OR "diagnostic system" OR "diagnostic system*" OR "diagnostic test" OR "diagnostic test" OR "diagnostic test*" OR "self-test" OR "self test*" OR "home-based test" OR "home-based test*" OR "self-sampling" OR "postal test*" OR "test kit*" OR "testing kit*" OR "tests kit*" OR "STI test*" OR "STD test*" OR "testing program*" OR "HIVST" OR "self-swabbing"):ti,ab,kw AND ("health information technology" OR "health information systems" OR "interactive health communication" OR "patient portal" OR "Telemedicine" OR "Telehealth" OR "web portal*" OR telemed* OR "ehealth" OR "e-health" OR "mhealth" OR "m-health" OR "mobile health" OR "telehealth" OR "tele-health" OR "tele health" OR "webbased" OR "web-based" OR "telemedicine" OR "tele-care" OR "telecare" OR "website" OR "websites" OR "webpage" OR "webpages" OR "web application" OR "web applications" OR "web access" OR "Internet" OR "internet" OR "online communication" OR "on-line communication" OR "on line communication" OR "text message*" OR "sms" OR "smart message service" OR "short message service"):ti) **OR** ("DirectLab" OR "swab2know" OR "getcheckedonline" OR "e-STI" OR "WeTest" OR "SELPHI" OR "eSexual" OR "chlamyweb"):ti,ab,kw**)**

**Academic Search Premier [full text search]**

http://search.ebscohost.com/login.aspx?authtype=ip,uid&profile=lumc&defaultdb=aph

(TI("Laboratory Technique" OR "Laboratory Techniques" OR "laboratory diagnosis" OR "laboratory diagnosis" OR "Clinical Laboratory Tests" OR "Laboratory Test" OR "Laboratory Testing" OR "lab test" OR "lab tests" OR "lab testing" OR "Laboratory Examination" OR "diagnostic tool" OR "diagnostic tool" OR "diagnostic assessment" OR "diagnostic assessment" OR "diagnostic system" OR "diagnostic system" OR "diagnostic test" OR "diagnostic test" OR "diagnostic test" OR "self-test" OR "self test" OR "home-based test" OR "home-based test" OR "self-sampling" OR "postal test" OR "test kit" OR "testing kit" OR "tests kit" OR "STI test" OR "STD test" OR "testing program" OR "HIVST" OR "self-swabbing") AND TI("health information technology" OR "health information systems" OR "interactive health communication" OR "patient portal" OR "Telemedicine" OR "Telehealth" OR "web portal" OR telemed OR "ehealth" OR "e-health" OR "mhealth" OR "m-health" OR "mobile health" OR "telehealth" OR "tele-health" OR "tele health" OR "webbased" OR "web-based" OR "telemedicine" OR "tele-care" OR "telecare" OR "website" OR "websites" OR "webpage" OR "webpages" OR "web application" OR "web applications" OR "web access" OR "Internet" OR "internet" OR "online communication" OR "on-line communication" OR "on line communication" OR "text message" OR "sms" OR "smart message service" OR "short message service") NOT TI("veterinary" OR "rabbit" OR "rabbits" OR "animal" OR "animals" OR "mouse" OR "mice" OR "rodent" OR "rodents" OR "rat" OR "rats" OR "pig" OR "pigs" OR "porcine" OR "horse" OR "horses" OR "equine" OR "cow" OR "cows" OR "bovine" OR "goat" OR "goats" OR "sheep" OR "ovine" OR "canine" OR "dog" OR "dogs" OR "feline" OR "cat" OR "cats")) **OR** TI("DirectLab" OR "swab2know" OR "getcheckedonline" OR "e-STI" OR "WeTest" OR "SELPHI" OR "eSexual" OR "chlamyweb") **OR** KW("DirectLab" OR "swab2know" OR "getcheckedonline" OR "e-STI" OR "WeTest" OR "SELPHI" OR "eSexual" OR "chlamyweb") **OR** SU("DirectLab" OR "swab2know" OR "getcheckedonline" OR "e-STI" OR "WeTest" OR "SELPHI" OR "eSexual" OR "chlamyweb") **OR** AB("DirectLab" OR "swab2know" OR "getcheckedonline" OR "e-STI" OR "WeTest" OR "SELPHI" OR "eSexual" OR "chlamyweb")**)**
