# Supplementary material for: Direct Access for Patients to Diagnostic Testing and Results Using eHealth: Systematic Review on eHealth and Diagnostics
Source: J Med Internet Res. 2022 Jan 12;24(1):e29303. doi: 10.2196/29303 (PMC8792777; doi:10.2196/29303)
Supplement: Multimedia Appendix 2 [file jmir_v24i1e29303_app2.docx]

**Appendix 2.** Mixed Method Appraisal Tool (MMAT)

In this table specific criteria per study design are described to assess the quality of study based on the MMAT.

|  | **Quality criteria** |
| --- | --- |
| **Study designs** |  |
| *Screening for all types* | 1.Are there clear research questions? |
|  | 2.Do the collected data allow to address the research questions? |
| Qualitative | 1.Is the qualitative approach appropriate to answer the research questions? |
|  | 2. Are the qualitative data collection methods adequate to address the research question? |
|  | 3. Are the findings adequately derived from the data? |
|  | 4. Is the interpretation of results sufficiently substantiated by data? |
|  | 5.Is there coherence between qualitative data sources, collection, analysis and interpretation? |
| Quantitative randomized controlled (trials) | 1.Is randomization appropriately performed? |
|  | 2.Are the groups comparable at baseline? |
|  | 3.Are there complete outcome data?^a^ |
|  | 4.Are outcome assessors blinded to the intervention provided? |
|  | 5.Did the participants adhere to assigned intervention? |
| Quantitative non randomized | 1.Are the participants representative of the target population?^b^ |
|  | 2.Are the measurements appropriate regarding both the outcome and intervention (or exposure)? |
|  | 3.Are there complete outcome data? ^a^ |
|  | 4.Are the confounders accounted for in the design and analysis? ^c^ |
|  | 5.During the study period, is the intervention administered (or exposure occurred) as intended?^d^ |
| Quantitative descriptive | 1.is the sampling strategy relevant to address the research question? |
|  | 2.Is the sample representative of the target population?^e^ |
|  | 3.Are the measurements appropriate? |
|  | 4.Is the risk of nonresponse bias low? |
|  | 5.IS the statistical analysis appropriate to answer the research question? |
| Mixed-methods | 1.Is there an adequate rationale for using a mixed methods design to address the research question? |
|  | 2.Are the different components of the study effectively integrated to answer the research question? |
|  | 3.Are the outputs of the integration of qualitative and quantitative components adequately interpreted? |
|  | 4.Are divergences and inconsistencies between quantitative and qualitative results adequately addressed? |
|  | 5.Do the different components of the study adhere to the quality criteria of each tradition of the methods involved? |

^a^ The study scored a ‘no’ when the attrition or dropout is higher or equal to 20% (23)

^b^ The study could have scored a ‘no’ for two reasons. First, when clear description of target population of target population and sample is given (by describing in and exclusion criteria), but reasons why people choose not to participate were not described. Second, the collected sample is not in line with target population (e.g., target population was 20-24 years old but a large proportion of sample is older than 24 years).

^c^ The study scored a ‘yes’ if age, ethnicity and sexual orientation is taken into consideration.

^d^ The study scored a ‘yes’ if the intervention or test kit was delivered in experimental group. The study scored a ‘no’ if the intervention or test kit was not properly delivered.

^e^ The study could have scored a ‘no’ for two reasons. First, clear description target population or sample is missing and reasons are not discussed for why eligible participants choose not to participate. Second, collected sample is not in line with target population (e.g., target population was 20-24 years old but a large proportion of sample is older than 24 years).
