# Supplementary material for: Direct Access for Patients to Diagnostic Testing and Results Using eHealth: Systematic Review on eHealth and Diagnostics
Source: J Med Internet Res. 2022 Jan 12;24(1):e29303. doi: 10.2196/29303 (PMC8792777; doi:10.2196/29303)
Supplement: Multimedia Appendix 3 [file jmir_v24i1e29303_app3.docx]

**Appendix 3**. Service provider characteristics per study

|  |  |  | Testing | | | | | Result | | | |
| --- | --- | --- | --- | --- | --- | --- | --- | --- | --- | --- | --- |
|  | Service name | Study^a^ | Disease(s) | Type of home-based test | Type specimen^b^ | Order, deliver, and return method^c^ | Instruction method | Method of notification | Independent HCP | Average delivery time in days | Linked to care or follow-up testing^d^ |
| **Triage service** | | |  |  |  |  |  |  |  |  |  |
|  | Fai il test anche TU project | Polilli et al. [12] | HIV, hepatitis B and C, syphilis |  |  |  |  |  |  |  |  |
| **Testing service** | | |  |  |  |  |  |  |  |  |  |
|  | C-project | Martin et al. [38] | Chlamydia | Self-sampling | Urine | O: Online  D: Postal service  R: In-person | ·∙ |  |  |  |  |
|  | Easy test | Jin et al. [34] | HIV | Self-testing | Blood | O: Online D: Postal service  R: n/a | Written |  |  |  |  |
|  | UCLA free HIV self-test program | Rosengren et al. [43] | HIV | Self-testing | Oral | O: Online  D: Postal service or pick-up  R: n/a | ·∙ |  |  |  |  |
|  | Social entrepreneurship testing | Zhong et al. [47] | HIV  Syphilis | Self-testing | Blood | O: Online  D: Postal service  R: Postal service^e^ | ·∙ |  |  |  |  |
|  | SELPHI | Witzel et al. [14] | HIV | Self-testing | Blood | O: Email  D: Postal service  R: n/a | Written, online videos |  |  |  |  |
|  |  | Witzel et al. [62] | HIV | Self-testing | Blood | O: Email  D: Postal service  R: n/a | Written, online videos |  |  |  |  |
|  | Unknown name | Andersen et al. [25] | Chlamydia | Self-sampling | Vaginal (F)  Urine (M) | O: Email or phone  D: Postal service  R: Post service | Written |  |  |  |  |
|  | Unknown name | Reagan et al. [58] | Chlamydia  Gonorrhea | Self-sampling | Urine | O: Phone  D: Postal service  R: Post service | Written |  |  |  |  |
|  | Unknown name | Grandahl et al. [48] | Chlamydia  Gonorrhea | Self-sampling | ·∙ | O: online  D: Postal service  R: ·∙ | ·∙ |  |  |  |  |
|  |  | Grandahl et al. [64] | Chlamydia  Gonorrhea | Self-sampling | ·∙ | O: online  D: Postal service  R: ·∙ | ·∙ |  |  |  |  |
| **Result service** | | |  |  |  |  |  |  |  |  |  |
|  | GxAlert | Babirye et al. [15] | Tuberculosis |  |  |  |  | SMS | Yes | ·∙ | Yes |
|  | Syfilistest.nl | Koekenbier et al. [35] | Syphilis |  |  |  |  | Online | Yes | 7 days | Yes |
|  | Early test | Morris et al. [39] | HIV |  |  |  |  | Online  Phone | Partly | 2-7 days | Yes |
|  | Result system of Denver Metro Health Clinic | Ling et al. [54] | Chlamydia, gonorrhea |  |  |  |  | Online | Partly | 7 days | Unclear |
|  | Excelleris | Mák et al. [55] | Not limited to a specific disease |  |  |  |  | Online | Yes | ·∙ | No |
|  | Patient portal | Talboom-Kamp et al. [50] | Not limited to a specific disease |  |  |  |  | Online | Partly | ·∙ | Yes |
|  | myCARE system | Robinson et al. [65] | Not limited to a specific disease |  |  |  |  | Online | Partly | ·∙ | Yes |
| **Triage & testing service** | | |  |  |  |  |  |  |  |  |  |
|  | A hora é Agora | De Boni et al. [27] | HIV | Self-testing | Oral | O: Online  D: Postal service or pick-up  R: n/a | Written |  |  |  |  |
|  | Online Chlamydia Testing program | Kwan et al. [36] | Chlamydia, gonorrhea | Self-sampling | Vaginal (F)  Urine (M) | O: Online  D: Pick-up service  R: In-person | Online |  |  |  |  |
|  | Swab2Know | Platteau et al. [41] | HIV | Self-sampling | Oral | O: Online  D: Postal service  R: Post service | Online video | Online  Email  Phone | Partly | 7 days | Yes |
|  | Don’t think, know | Rotblatt et al. [44] | Chlamydia, gonorrhea | Self-sampling | Vaginal | O: Online, phone  D: Postal service  R: Post service | Written, online video | Online  Phone | Partly | 7 days | Yes |
|  | Testikodus | Rüütel et al. [45] | Chlamydia, gonorrhea, trichomonas, LGV, myco-plasmosis | Self-sampling | Urine | O: Online  D: Postal service  R: Post service, in-person | ·∙ | Online | Yes | 5 days | Yes |
|  | RUClear | Ahmed-Little et al. [61] | HIV | Self-sampling | Blood | O: Online  D: Postal service  R: Post service | Written, online video | Phone  SMS  Letter | Partly | ·∙ | Yes |
| **Triage, testing & result service** | | |  |  |  |  |  |  |  |  |  |
|  | DS@H | Elliot et al. [28] | HIV | Self-sampling | Oral, blood | O: Online  D: Postal service  R: Post service | Written | SMS  Phone | Partly | 1 day after sample received | Yes |
|  | GetCheckedOnline | Gilbert et al. [13] | Chlamydia, gonorrhea | Self-sampling | Urine, oral, rectal | O: Online  D: Pick-up  R: In-person | Written | Online  Phone | Partly | 7-14 days | Yes |
|  |  | Gilbert et al. [52] | Chlamydia, gonorrhea | Self-sampling | Urine, oral, rectal | O: Online  D: Pick-up  R: In-person | Written | Online  Phone | Partly | 7-14 days | Yes |
|  |  | Knight et al. [63] | Chlamydia, gonorrhea | Self-sampling | Urine, oral, rectal | O: Online  D: Pick-up  R: In-person | Written | Online  Phone | Partly | 7-14 days | Yes |
|  |  | Dulai et al. [49] | Chlamydia, gonorrhea | Self-sampling | Urine, oral, rectal | O: Online  D: Pick-up  R: In-person | Written | Online  Phone | Partly | 7-14 days | Yes |
|  | Let’s talk about it NHS | Nadarzynski et al. [40] | Chlamydia, gonorrhea, HIV, syphilis, hepatitis B and C | Self-sampling | Vaginal (C&G), blood (S, H, HepB/C) | O: Online, phone  D: Postal service  R: Post service | Written, online video | SMS  Phone | Partly | 7 days | Yes |
|  | Checking In | Ricca et al. [42] | HIV | Self-sampling | Blood | O: Online  D: Postal service  R: Post service | ·∙ | Phone | Partly | 7 days | Yes |
|  | eSTI | Spielberg et al. [46] | Chlamydia, gonorrhea, trichomonas | Self-sampling | Vaginal | O: Online  D: Postal service  R: Post service | ·∙ | Online | Yes | ·∙ | Yes |
|  | SH:24 | Barnard et al. [51] | Chlamydia, gonorrhea, HIV, syphilis | Self-sampling | Blood (S&H), vaginal (C&G in F), urine (C&G in M), and oral, rectal, and urine (C&G in MSM) | O: Online  D: Postal service  R: Post service | Written, online video | SMS  Phone | Partly | 7 days | Yes |
|  |  | Wilson et al. [59] | Chlamydia, gonorrhea, HIV, syphilis | Self-sampling | Blood (S&H), vaginal (C&G in F), urine (C&G in M), and oral, rectal, and urine (C&G in MSM) | O: Online  D: Postal service  R: Post service | Written, online video | SMS  Phone | Partly | 7 days | Yes |
|  |  | Wilson et al. [60] | Chlamydia, gonorrhea, HIV, syphilis | Self-sampling | Blood (S&H), vaginal (C&G in F), urine (C&G in M), and oral, rectal, and urine (C&G in MSM) | O: Online  D: Postal service  R: Post service | Written, online video | SMS  Phone | Partly | 7 days | Yes |
|  | Freetesting.hiv | Brown et al. [56] | HIV | Self-sampling | Blood | O: Online  D: Postal service  R: Post service | Written, online video | SMS  Phone | Partly | 7 days | Yes |
|  | Chlamyweb | Kersaudy-Rahib et al. [57] | Chlamydia | Self-sampling | Vaginal (F), urine (M) | O: Online  D: Postal service  R: Post service | ·∙ | Email  Post | Partly | ·∙ | Yes |
|  | I Want The Kit^f^ | Chai et al. [26] | Chlamydia, gonorrhea, trichomonas | Self-sampling | Penile, urine | O: Online  D: Postal service  R: Post service | Written | Phone | No | ·∙ | Yes |
|  |  | Gaydos et al. [31] | Chlamydia, gonorrhea, trichomonas | Self-sampling | Vaginal | O: Online, phone  D: Postal service, pick-up  R: Postal service | Written | ·∙ | ·∙ | ·∙ | ·∙ |
|  |  | Gaydos et al. [32] | Chlamydia | Self-sampling | Vaginal | O: Online, phone  D: Postal service, pick-up  R: Postal service | Written | ·∙ | ·∙ | ·∙ | ·∙ |
|  |  | Gaydos et al. [33] | Chlamydia | Self-sampling | Vaginal | O: Online, phone  D: Postal service, pick-up  R: Postal service | Written | Phone | No | 14 days | Yes |
|  |  | Gaydos et al. [29] | Chlamydia, gonorrhea, trichomonas | Self-sampling | Urogenital, rectal | O: Online  D: Postal service  R: Postal service | Written | ·∙ | ·∙ | ·∙ | ·∙ |
|  |  | Gaydos et al. [30] | Trichomonas | Self-testing | Vaginal | O: Online  D: Postal service  R: Postal service^g^ | Written | ·∙ | ·∙ | ·∙ | Unclear |
|  |  | Kuder et al. [53] | Chlamydia, gonorrhea, trichomonas | Self-sampling | Urogenital, rectal | O: Online, phone  D: Postal service  R: Postal service | Written | Online | Yes | ·∙ | Yes |
|  |  | Ladd et al. [37] | Chlamydia, gonorrhea, trichomonas | Self-sampling | Rectal, vaginal | O: Online, phone  D: Postal service  R: Postal service | Written | SMS  Email  Phone  Letter | Unclear | ·∙ | ·∙ |

**Note.** HCP= health care professional. HIV = human immunodeficiency virus. SMS = short message service. MSM = men having sex with men. C&G = Chlamydia and Gonorrhea. S&H= Syphilis and human immunodeficiency virus. LGV = Lymphogranuloma venereum. ·∙= missing info

^a^ Identifies the first author and publication year of the study examining the respective service.

^b^ If the required type of specimen differed between sexes, an F behind the specimen indicated that it was for women and an M indicated it was for males. When a provider offered testing for different diseases, the required specimen per disease was specified.

^c^ Identifies the method used for ordering (O), delivering (D) and receiving (R) the test kit.

^d^ Identifies whether the individual is directly linked to care or to follow-up testing.

^e^ If participants returned the test results to the laboratory, participants were refunded the money for the test.

^f^ The service was listed as triage, testing and result service; however, the communication of test results was not independent from a health care professional in each of the studies that examined the service or it was unclear whether the communication of test results was independent from a health care professional.

^g^ Returning the test kit was optional.
