# Supplementary material for: Direct Access for Patients to Diagnostic Testing and Results Using eHealth: Systematic Review on eHealth and Diagnostics
Source: J Med Internet Res. 2022 Jan 12;24(1):e29303. doi: 10.2196/29303 (PMC8792777; doi:10.2196/29303)
Supplement: Multimedia Appendix 4 [file jmir_v24i1e29303_app4.docx]

**Appendix 4.** Overview of the reported outcomes for the triage, testing, and result services

|  | Outcome | Study | Service^a^ | Results |
| --- | --- | --- | --- | --- |
| **Triage service** | |  |  |  |
|  | Usage | Polilli et al. [12] | Tr | About 6000 users visited the website, a little over 5000 users also completed the risk assessment, and nearly 3500 made an appointment for clinic-based testing. |
|  | Linkage to testing or treatment | Polilli et al. [12] | Tr | A total 3500 users scheduled a clinic-based test and 87% was present for testing. In total, 28 individuals (<1%) were tested positive for HIV, and 93% were linked to care. |
|  | Predictive value of risk assessment | Gaydos et al. [29] | Tr, Te, Re | In females, a higher risk assessment score predicted the risk for having a STI, independent of age and race. In males, the risk score did not significantly predict STI after controlling for race. |
|  |  |  |  |  |
| **Testing service** | |  |  |  |
|  | Acceptability/ usability | Ahmed-Little et al. [61] | Tr, Te | Among 2563 respondents, 97% (strongly) agreed that it was acceptable to order a HIV test kit online, 80% felt that this method of testing was easily accessible, 94% found the test instructions easy to understand. |
|  |  | Chai et al. [26] | Tr, Te, Re | Among the 476 respondents, 75% preferred a self-test versus a clinic-based test, 85% believed it was safe, 88% found it (very) easy to use, and 89% would use internet-screening again. |
|  |  | De Boni et al. [27] | Tr, Te | Among the 362 respondents, 91% found the website (very) easy to use, 72% had no difficulties navigating the website, 6% could not find the pages they were searching for, and 94% found the instructions for testing clear. |
|  |  | Gaydos et al. [30] | Tr, Te, Re | Among the 102 respondents, 84% found it easy to follow the instructions for the STI test kit, 95% found it easy collect the specimen, 90% found it easy to interpret the results, and 97% (somewhat) trusted the results. Moreover, 62% preferred self-testing, 77% would recommend the test to a friend, and 80% would get the test if it were available of the counter. |
|  |  | Gaydos et al. [32] | Tr, Te, Re | Of the 1171 respondents, 92% would use this program for STI tests kits again, 91% preferred self-collection specimens over the clinic, and 97% reported that it was (very) easy to use the kit. |
|  |  | Gaydos et al. [33] | Tr, Te, Re | Of the 400 respondents, 89% preferred to collect a self-administered test sample, 88% believed it was safe, 90% thought it was (very) easy to use, 86% would use this method of online ordering and home testing again, and 73% preferred at-home delivery of the test kit compared to a pharmacy pickup. |
|  |  | Knight et al. [63] | Tr, Te, Re | A total of 37 users of an online diagnostic service participated in qualitative interviews. Most users had a preference for this platform instead of clinic-based testing, because of convenience, privacy, and control over specimen collection. Users preferred receiving their results online via the platform compared to phone or email by a clinic staff. |
|  |  | Kwan et al. [36] | Tr, Te | Among the 55 respondents, 96% reported that the Chlamydia testing program was easy to use and 98% would recommend it to a friend. |
|  |  | Reagan et al. [58] | Te | Among the 129 respondents, 95% found it easy to follow the instructions for the STI test kit. |
|  |  | Rosengren et al. [43] | Te | Among the 56 respondents, 93% found the HIV test kit (very) easy to use and 74% (somewhat) preferred the test kit over clinical testing. |
|  |  | Spielberg et al.[46] | Tr, Te, Re | Among the 106 respondents, 95% prefer the online over clinical testing, 80% would rather test online than go to the clinic for future testing, and 98% would recommend the study to a friend. |
|  |  | Wilson et al. [60] | Tr, Te, Re | Of the 84 respondents of the group that did online STI testing, 75% found the process of online ordering a STI test, at-home sampling and receiving results online acceptable. |
|  |  | Wilson et al. [59] | Tr, Te, Re | Among 294 respondents of the group that did online STI testing, 71% found the process of online ordering a STI test, at-home sampling, and receiving the result via a SMS acceptable. |
|  |  | Witzel et al. [14] | Te | Among the 375 respondents, 98% found the instructions of the HIV test kit easy to understand, 97% found the test kit simple to use and 97% reported an overall good experience. |
|  |  | Dulai et al. [49] | Tr, Te, Re | Among the 1255 respondents who filled in the survey at this question, 33.1% reported that they found it easier to go to the clinic. Among those who used the service, 44 respondents of 1268, 90% would use the service again. |
|  |  | Grandahl et al. [48] | Te, Re | Among the 1785 respondents, more than 90% found the service good or very good. |
|  |  | Grandahl et al. [64] | Te, Re | A total of 20 users of an online diagnostic service participated in qualitative interviews. Most users highly appreciated the service and found the service easy to use, convenient and confidential. Some barriers they mentioned were language, uncertainty about the procedure, unreliable postal service and insecure handling of data. They would use the service again in the future, even if it was against some costs. |
|  | Cost-effectiveness | Ahmed-Little et al. [61] | Tr, Te | Overall costs for testing, per person, were £31-£32 (€26.47-€27.32), which is in line with testing costs in national HIV testing pilots. |
|  |  | Kersaudy-Rahib et al. [57] | Tr, Te, Re | Home-based self-sampling for chlamydia was €32 per kit compared to €73 for clinical testing. The costs per positive tests were three times higher in the STI clinics than for home tests (€1123 versus €375). |
|  | Usage | Ahmed-Little et al. [61] | Te | In total 5179 HIV test kits that were send out and 59% were returned. |
|  |  | Andersen et al. [25] | Te | The website was visited by 651 people of whom 9% (*n* = 60) ordered a chlamydia test kit online. Another 309 ordered a kit through an answering machine. In the end, 342 were sent a kit and 68% submitted a sample for analysis. |
|  |  | Barnard et al. [51] | Tr, Te, Re | A STI kit was ordered by 3515 people whom 73% returned a sufficient sample. |
|  |  | Brown et al. [56] | Tr, Te, Re | Of the 8999 who ordered a HIV self-sampling kit, 54% returned their kit. Kit return was highest in the group that received a primer text message prior to the kit’s arrival and additional behavioral insight reminders (56% of *n* = 2267). |
|  |  | Chai et al. [26] | Tr, Te, Re | In total 1644 STI home tests were ordered online, and 31% of the ordered tests were returned of which 98% as analyzed. |
|  |  | De Boni et al. [27] | Tr, Te | The website had 17.786 unique visitors, 3885 HIV self-test kits were ordered of which 65% received the test. 21% of the users returned the self-test result online. |
|  |  | Elliot et al. [28] | Tr, Te, Re | In total 17361 people completed the online risk assessment for a HIV self-sampling kit, 59% ordered a kit (*n* = 10.323). 55% returned the sample for analyses. |
|  |  | Gaydos et al. [31] | Tr, Te, Re | In total, 3774 people ordered a chlamydia self-sampling test and 32% also returned the sample. |
|  |  | Gilbert et al. [13] | Tr, Te, Re | In total 868 users created an account at the testing service, of whom 318 users submitted specimens (37%). |
|  |  | Kersaudy-Rahib et al. [57] | Tr, Te, Re | In the group (*n* = 5531) offered a chlamydia self-sampling test, 2616 users received the test of whom 1616 returned the sample (62%). Test rates were higher in the group that received a self-sampling kit compared to the group that was invited to be screened in primary care (29 vs 9%). |
|  |  | Kuder et al. [53] | Tr, Te, Re | The kit return rate was 62% (691/1116) before an automated test-result service was implemented and was 66% (858/1303) after implementation. The experimental group (n=1303), after web design changes to order a STI test kit, 62% used the test. The control group (n=1116), before website design changes, 66% used the test. |
|  |  | Kwan et al. [36] | Tr, Te | In total, 675 users completed an online risk assessment and requested a chlamydia self-sampling test. A total of 377 tests were performed (56%). |
|  |  | Ladd et al. [37] | Tr, Te, Re | In total, 406 people ordered a test kit for rectal STIs of whom 51% returned specimen. |
|  |  | Martin et al. [38] | Te | 413 chlamydia test kits were ordered (48% via email, 49% during outreach events, 2% via phone). 195 samples (43%) were returned. |
|  |  | Reagan et al. [58] | Te | STI kit return rate was higher in the group that received the self-sampling kit at home compared to those who received it at a clinic (resp. 72 vs 48%). |
|  |  | Ricca et al. [42] | Tr, Te, Re | In total 896 users received a HIV self-sampling test kit, 82% returned the specimen for analysis. |
|  |  | Rosengren et al. [43] | Te | The website received 4389 unique visitors and resulted in 333 request for a HIV test kit. In the group who completed the follow-up survey (*n* = 56), everyone used the kit. |
|  |  | Rotblatt et al. [44] | Tr, Te | In total 2927 STI self-sampling kits were ordered online or via phone, of which 55% returned their specimen for analyses. The majority (95%) of the specimen were usable for analyses. |
|  |  | Rüütel et al. [45] | Tr, Te | 24% of those who ordered a self-sampling STI test, provided the specimen (65/265). |
|  |  | Spielberg et al. [46] | Tr, Te, Re | In total 213 people ordered a STI self-sampling test kit, 67% returned the specimen. |
|  |  | Wilson et al. [60] | Tr, Te, Re | People who were willing to do an STI test were randomized to receive a text message with referring them to a website with either an online test service or a list with the locations of clinics. In the group referred to online testing, the test rate was significantly higher (45 vs 24%) and there was a reduced time to test (29 vs 36 days). |
|  |  | Wilson et al. [59] | Tr, Te, Re | In the group(*n* = 921) who received home tests and the results online, 50% did the STI test in comparison with 27% in the group(*n* = 818) who were offered a test and results at the clinic. This was a significant difference. The time from randomization in to the groups to completion of the STI test was shorter in the group who did home testing(28.8 days versus 36.5 days). This was a significant difference. The median time from diagnosis to treatment was for the group who did home testing group 2 days and for the control group 4 days |
|  |  | Witzel et al. [14] | Te | In total 631 users were sent a HIV test kit. In the two-week follow-up survey, completed by *n* = 405, 95% indicated having received the kit and 83% had used it. People had not used it, if they planned to use it later (97%) or had tested elsewhere (3%). |
|  |  | Zhong et al. [47] | Te | 198 HIV self-test kits were ordered online, and 192 provided feedback. Of these, 178 (93%) had used the test. |
|  |  | Grandahl et al. [48] | Te, Re | Among the 1785 users, 85% returned their sample. Reasons for not returning the kit were lack of time(22.5%) or they were no longer worried (12.1%). |
|  | Other outcomes | Martin et al. [38] | Te | Among the 195 respondents, a common reason for requesting a chlamydia test kit was that it was for free (49%) and easy to get (39%). |
|  |  | Witzel et al. [14] | Te | Motivations of users to do HIV self-testing: (a) reduced HIV testing barriers (opportunity barriers such as convenience and ease of use and motivational barriers like confidentiality and stigma), (b) desire to use new technology, and (c) altruistic motivation. |
|  |  | Zhong et al. [47] | Te | Among the 198 respondents, the two main reasons for doing a HIV self-test were convenience and to save time (46%), and privacy (40%). Facilitators to use the purchased HIV self-test were anonymity (56%), ease of use (49%), and ability to test alone (41%). Barriers were accuracy (43%), potential costs (40%), and concern about having to interpret the results (36%). Besides, 67% of the respondents reported that they would use the test kits in the future if it was free. |
|  |  | Dulai et al. [49] | Tr,Te,Re | Among the 1247 respondents who filled in this question at the survey, 20 percent was worried the *privacy* of his online information and 5% had low trust in this service. |
|  |  | Witzel et al. [62] | Te | Reasons for using HIV self-testing by trans men and women were inaccessible and inappropriate clinical services. |
|  |  |  |  |  |
| **Result service** | |  |  |  |
|  | Usage | Gilbert et al. [52] | Tr, Te, Re | Users of the online platform had significant shorter waiting time for the test results than clinic clients (respectively 1% versus 12% was still waiting on the test results at the time of the survey) |
|  |  | Koekenbier et al. [35] | Re | Of all the 93 users, 97% obtained their results from the website |
|  |  | Ling et al. [54] | Re | Test result retrieval was assessed in three periods. In period 1 (n=3624) results were communicated over the phone. In period 2 (*n* = 3931), online results were optional and 41% opted to receive their results online. In period 3 (*n* = 1501), online results was the standard. In period 2 and 3, significantly more users received their results in the group that opted for online results compared to the group that opted to receive their results over the phone. 74% of those in period 2 and 3 who opted or accepted online results also accessed their results. Yet the overall proportion of users who received their results was not significantly different before or after the online result option became available. The number of users who called for results did decrease significantly from period 1 (67%) to period 3 (36%). |
|  |  | Morris et al. [39] | Re | Among the 3070 users, 69% obtained their results either by the Internet or via an automated voicemail service. Of this group, 65% used the Internet to look up their result. |
|  |  | Platteau et al. [41] | Tr, Te | In total 1071 tests were done, 99% of results (1057/1071) were delivered through the website. Significantly more users collected their test results when the test was ordered online compared to tests performed during outreach activities (98% vs 87%). |
|  |  | Rotblatt et al. [44] | Tr, Te | In total 1619 tests were done, 88% of the users retrieved their results on the website. |
|  |  | Spielberg et al. [46] | Tr, Te, Re | In total 143 tests were done, of those 92% viewed their results online, with 88% who viewed their results the same day they were posted. |
|  | Comprehension | Babirye et al.[15] | Re | In total 123 respondents were reached via text message, 93% comprehended the text message with test result. |
|  |  | Mák et al. [55] | Re | Among the 1852 respondents, 75% who had online access to their test results was confident that they fully understood the results. Among the 1119 respondents who had no online access to their results, the comprehension was significantly higher, namely 85%. |
|  |  | Robinson et al. [65] | Re | Among the 21 respondents the comprehension of the results differed from difficulty with understanding of the results to no difficulty. However, users who mentioned that they had difficulties with understanding the result described that the reference range of their result was very helpful and this made it easier to use. |
|  |  | Nadarzynski et al. [40] | Tr, Te, Re | Among the 100 respondents, 13% who received a text message with their results indicated that they did not understand its content, 92% of the respondents understood that no further action is required when a text message states that the STI test is “negative (clear)”. |
|  | Acceptability | Ling et al.[54] | Re | In total 429 respondents filled in the questionnaire. Two main reason for opting for online results was (a) that test results could be accessed any time of the day (75%) and (b) because respondents believed that they would receive their results faster (37%). Two main reasons for declining online results was (a) that respondents preferred to call the clinic (43%) and (b) because of limited access to the internet (32%). |
|  |  | Morris et al. [39] | Re | Among the 235 respondents, 87% was (very) comfortable with receiving their results online. |
|  |  | Platteau et al. [41] | Tr  Te | Among the 388 respondents, 96% were very satisfied with the process of ordering a test kit online and receiving the results online, 4% reported mixed feelings. |
|  |  | Spielberg et al. [46] |  | Among the 106 respondents, 98% thought the website – that offered triage, testing and result service - was (very) easy to use. |
|  | Other outcomes | Babirye et al. [15] | Re | In Uganda, 233 users were eligible to receive an SMS with their tuberculosis test result. 152 of these users (correctly) entered their phone number (65%), with 145 messages being transmitted by the local SMS service provider to the phone network (95%). A total of 123 users were contacted of whom 93% confirmed having received the result. |
|  |  | Mák et al. [55] | Re | Group 1 (*n* = 1856) received test results online, 84% of those received their results within a few days. Group 2 (*n* = 1087) had not online access to their results, 38% of those received their results in a few days. This difference in wait time was significant.  Of 2990 questionnaire respondents, 86% reported no/low anxiety after results. This level differed not between users with online access versus those who had not. |
|  |  | Nadarzynski et al. [40] | Tr, Te, Re | Of the 115 questionnaire respondents, 86% preferred text messages that included the names of the tested STI and that included the results of all STIs tested (in one message). |
|  |  | Robinson et al. [65] | Re | Users felt feel more comfort and engaged with their healthcare when they see the results themselves. This service is not limited to a specific disease. Besides they reported that it had no negative effects on themselves, seeing the results. Besides, they thought that it would lead to better communication with the HCP.  Reasons for the users to use this online result portal were this better communication, convenience and being a steward of own healthcare. |
|  |  | Talboom-Kamp et al. [50] | Re | The questionnaire was completed by 354 of 13907 patients who viewed their results of their laboratory test online (not limited to a specific disease). In this questionnaire the Information and Presentation score was measured and scored a 67.70 (SD 13.12) and the mean Motivation and  Confidence to Act score was 63.59 (SD 16.22). Those are two subscales of the eHIQ. These results showed that users found online viewing results easy to use, trustworthy and appropriate. The self-efficacy of users was also relatively high according to this score, but below the cutoff score for a positive attitude. |
| **Test and result services** | |  |  |  |
|  | Follow up testing and treatment^b^ | Ahmed-Little et al. [61] | Tr, Te | 3062 tests were done, seven infections were detected, and 100% did confirmatory testing. |
|  |  | Chai et al. [26] | Tr, Te, Re | 501 tests were done, 21% was tested positive, and 99% of those tested positive were treated. |
|  |  | De Boni et al. [27] | Tr, Te | Among the 542 tests, 34 (6%) were positive or invalid. In total 37 users did a confirmatory tests: 30 were positive. |
|  |  | Elliot et al. [28] | Tr, Te, Re | Among the 5249 unique tests, 2% tested positive for HIV. Of those, 67% were confirmed as new HIV diagnosis and received treatment, 11% were false reactive, 11% already diagnosed with HIV, and for 10% treatment was unconfirmed. |
|  |  | Gaydos et al. [32] | Tr, Te, Re | Among the 1171 users, four (0.3%) had a positive test and all were treated. |
|  |  | Gaydos et al. [31] | Tr, Te, Re | Among the 1203 tests, 9.1% was tested positive for chlamydia of which 96.5% was treated. Among the 496 tests for gonorrhea or trichomonas, everyone with a positive test for gonorrhea (3%) or trichomonas (7%) were treated. |
|  |  | Jin et al. [34] | Te | Of the 879 people who were eligible for HIV self-testing and received the test kit. Among the 683 returned a photograph of the test result, 98(14%) had a positive test result and of those 72% received treatment. |
|  |  | Kersaudy-Rahib et al. [57] | Tr, Te, Re | In the group (*n* = 5531) who received their chlamydia test kit at home 110 (7%) was tested positive and 91% visited a doctor. In the group who were invited to test in primary care setting, 30(6%) tested positive and 87% had seen a doctor. |
|  |  | Koekenbier et al. [35] | Re | Among the 93 tests, 15% tested positive and, of those, 71% did confirmatory testing. |
|  |  | Kuder et al. [53] | Tr, Te, Re | In the group that needed to call for their test result (n = 691), 11% tested positive and 58% of the positive tests received treatment. In the group( n= 858), with an website with automated test result service, 10% tested positive and 87% of the positive users received treatment |
|  |  | Kwan et al. [36] | Tr, Te | Among the 377 tests, 18% of the users tested positive for chlamydia or gonorrhea and 100% of the positive tested users were treated and 94% was treated within 14 days. |
|  |  | Ling et al. [54] | Re | When results were communicated over the phone (*n* = 193), 81% confirmed treatment within 30 days. When online results were optional (*n* = 240), 82% confirmed treatment and, when online results were the norm (*n* = 110), 71% confirmed treatment. The difference was not significant. |
|  |  | Platteau et al. [41] | Tr, Te | Among the 1071 tests, 2% were HIV positive and 100% of the positive tested users were linked to care. |
|  |  | Ricca et al. [42] | Tr, Te, Re | Among the 735 tests, 25 users tested positive for HIV (3%) 14 sought care, three had not and for eight it is unknown. |
|  |  | Rosengren et al. [43] | Te | 56 users reported the results of the HIV self-test (45%), of whom 4% tested positive. All positive tested users did confirmatory testing and had medical care. |
|  |  | Rotblatt et al. [44] | Tr, Te | Among the 1619 tests, 8% tested positive for a STI. For 88% of the positive users, treatment was confirmed. |
|  |  | Spielberg et al. [46] | Tr, Te, Re | Among the 143 tests, 6% were tested positive and all received treatment. |
|  |  | Zhong et al. [47] | Te | Among the 178 tests, six tests were positive for STI and 8 were positive for HIV. All did confirmatory testing, where seven HIV positive results were confirmed and all received treatment. |

**Note.** STI = sexually transmitted infection. HIV = human immunodeficiency virus. SMS = short message service.

^a^ The abbreviations highlight the type of service: Tr= Triage service, Te= Test service, Re= Result service.

^b^ Data related to follow up testing and treatment is discussed simultaneously testing and result services to avoid overlap because it referred to both outcome measures.
